# Dr. Bennet Dowler

**Published:** 1853-05

**Authors:** 


*Dr. Bennet Dowler.*—The friends of this distinguished experimentalist and writer, will be gratified to learn of a flattering distinction which his labors have procured. The Royal Society of Northern Antiquarians, of Copenhagen, of which the King of Denmark is president, have signified their intention of electing him a fellow. We congratulate him most cordially on this new honor.

